# Experiments with Amalgam

**Published:** 1893-09

**Authors:** 


ARTICLE IV.

### EXPERIMENTS WITH AMALGAM.

BY B.

I have been experimenting for some time on amalgams. First, I put into nitric acid, one part to four of water, a lump of hard amalgam; second, hydrochloric acid one part to two of water; third, sulphuric acid one part to two-fourths strong vinegar. A lump of dry amalgam has remained in each since yesterday; the only effect noticeable on either is a slight action of the nitric acid darkening slightly the surface without any perceptible change; none of the others have undergone any perceptible change, but remain clear and white.

Now, any of these preparations are sufficiently strong to act with energy on teeth in the same length of time, and any of these acids would, if retained in the mouth any length of time, excoriate the entire mucous surfaces. Good amalgams are composed of pure tin and silver, and amalgamated with pure mercury. Water does not decompose mercury, silver or tin to any perceptible extent; nitric acid dilute acts on silver, also mercury, separately, and less so on tin, heat facilitating the action; when the three are combined, as in amalgam, the acid action is greatly lessened. I do not believe that any action of the fluids of the mouth is sufficient to produce any mercurial salt capable of acting injuriously to the slightest extent, even in cases that have been repeatedly salivated by taking mercurials; if so, I have never witnessed a case during many years professional observation.

I have seen a filling, but a short time since, made of silver filings and mercury, that had been in a lower bicuspid twenty-five years, the filling being perfectly sound, and the tooth all round, except near the gum, where a cavity below had nearly reached the filling. This filling was very dark on the surface, but on running a file over it slightly, it gave a pure, sound white surface; in consequence I left the filling in, and filled below it. This dark surface was the result of the silver oxidizing slightly.

Remove any amalgam filling from any tooth, and file the surface, and the filed portion will become white and metallic. In order to get protoxide of mercury, which is the only one of consequence, mercury must be heated up to 600 degrees with free access of air; then red precipitate is formed, which is the protoxide, and on raising the heat higher, this oxide is again decomposed into the simple elements.

To form calomel, which is a subchloride, subnitrate of mercury is precipitated by common salt; it is also formed by other processes. Protochloride of mercury, or corrosive sublimate, may be made in several ways. When metallic mercury is heated in chlorine gas, it takes fire and burns, producing this salt.

From the above formulas it will be seen that mercury is not readily acted on by any fluids that may exist in the mouth, as these fluids always contain, at least, from 800 to 900 parts of water in 1,000 parts, so that any acid or any other agent contained in this fluid could absolutely have no action of any moment, either on tin or silver. The latter turns dark from an oxide being formed in some mouths much more readily than in others, some mouths scarcely acting on a silver plate at all. Zauman says that mercury slowly vaporizes at all temperatures above forty degrees; some say all temperatures above sixty six. The vaporization goes on more rapidly as the temperature is raised up to the point of ebullition 662.

All the apprehension that need give us any concern in connection with amalgam fillings, is the vaporization during the process of hardening, some of which undoubtedly will be inhaled into the lungs, as this vapor must be lighter than air or it could not be a vapor at all.

The amount that might be inhaled would be so insignificant that it would not do any mischief, as it would be carried out of the lungs again, even if it passed the entire rounds of the circulation.

Workers in quicksilver are short-lived, owing to the fact that they are constantly in an atmosphere charged with these vapors, which, no doubt, keeps their systems saturated during their working hours, which in a few years causes a total lesion of nutrition; the hair and nails fall off, the hard tissues become saturated, the perosteum fails to nourish the bones, and the poor wretches die from exhaustion.

The insignificant amount of this vapor escaping from a fine amalgam filling could produce no injurious effect. The vapors will salivate when sufficient has been inhaled, which is the first effect of almost all forms of mercury, however introduced into the system. Mercury, in its action, is an irritating stimulant to the glands, more especially the liver primarily and the oral secondarily.

All the specimens have now been in the acids forty-eight hours. None of them are in the least affected, except that in the nitric acid, which is nearly all decomposed, with some precipitate of tin, I suppose, at the bottom. It will be seen that in twenty-four hours there was scarcely any action at all by the nitric acid, and now none at all by any of the other acids. I think these conclusions are sustained by demonstrable facts as given above.—*Dominion Dental Journal.*

